# Enteroviral Infections in the First Three Months of Life

**DOI:** 10.3390/pathogens11010060

**Published:** 2022-01-03

**Authors:** Marcello Sandoni, Lidia Ciardo, Caterina Tamburini, Alessandra Boncompagni, Cecilia Rossi, Isotta Guidotti, Elisabetta Garetti, Licia Lugli, Lorenzo Iughetti, Alberto Berardi

**Affiliations:** 1 Pediatric Post-Graduate School, University of Modena and Reggio Emilia, 41125 Modena, Italy; marcello.sandoni@gmail.com (M.S.); lidiaciardo@libero.it (L.C.); cate.tamburini@gmail.com (C.T.); lorenzo.lughetti@unimore.it (L.I.); 2 Neonatal Intensive Care Unit, Women’s and Children’s Health Department, Azienda Ospedaliera, University of Modena and Reggio Emilia, 41125 Modena, Italy; boncompagni.alessandra@aou.mo.it (A.B.); rossi.cecilia@aou.mo.it (C.R.); guidotti.isotta@aou.mo.it (I.G.); garetti.elisabetta@aou.mo.it (E.G.); alberto.berardi@unimore.it (A.B.); 3 Pediatric Unit, Women’s and Children’s Health Department, Azienda Ospedaliera, University of Modena and Reggio Emilia, 41125 Modena, Italy

**Keywords:** enterovirus, neonate, infant, infection, treatment

## Abstract

Enteroviruses (EVs) are an important source of infection in the paediatric age, with most cases concerning the neonatal age and early infancy. Molecular epidemiology is crucial to understand the circulation of main serotypes in a specific area and period due to their extreme epidemiological variability. The diagnosis of EVs infection currently relies on the detection of EVs RNA in biological samples (usually cerebrospinal fluid and plasma, but also throat swabs and feces) through a polymerase chain reaction assay. Although EVs infections usually have a benign course, they sometimes become life threatening, especially when symptoms develop in the first few days of life. Mortality is primarily associated with myocarditis, acute hepatitis, and multi-organ failure. Neurodevelopmental sequelae have been reported following severe infections with central nervous system involvement. Unfortunately, at present, the treatment of EVs infections is mainly supportive. The use of specific antiviral agents in severe neonatal infections has been reported in single cases or studies including few neonates. Therefore, further studies are needed to confirm the efficacy of these drugs in clinical practice.

## 1. Introduction

Enteroviruses (EVs) are small single-stranded, positive sense RNA viruses belonging to the family *Picornaviridae*. This family also includes the genera Parechovirus and Hepatovirus. The genus Enterovirus also encompasses the species Rhinovirus A, B, and C. Thanks to molecular typing methods, more than one hundred EVs serotypes are known to infect humans and have been recently reclassified into four species (from A to D), as reported in Table 1 [1,2]. 

In micrographs, the viruses are 27- to 30-nm particles and show icosahedral non-enveloped capsids and dense cores containing the genomic RNA, which is approximately 7.5 kb in length. Enteroviral infection of the cell begins with the interaction of the virion with one or more cell surface protein and receptors, which are usually represented by integrins or immunoglobuline-like proteins. These receptors have been identified for some serotypes. For example, coxsackievirus B1–6 are known to interact with the coxsackie and adenovirus receptor (CAR) and some coxsackievirus A serotypes use the intercellular adhesion molecule 1 (ICAM-1). Following attachment of the virus, recruitment of additional cellular receptors occurs, and the virion is enveloped by cell membrane. A steric shift in the capsid conformation occurs, resulting in uncoating and release of RNA freely into the cellular cytoplasm, where it rapidly binds to ribosomes and begins protein synthesis. A single polypeptide is produced, which is almost instantaneously auto-cleaved by viral proteases. This polyprotein contains three domains, P1 to P3. The structural protein VP1 to VP4 originates from the cleavage of the P1 domain. Portions of VP1, VP2 and VP3 are exposed on the surface of the capsid, whereas VP4 is completely internal. The replication of enteroviruses occurs in the cytoplasm and is completed rapidly in 5–10 h [3]. 

Phylogenetic analysis has revealed that circulating EVs have high mutation rates and frequently undergo recombination, leading to increased genetic diversity, which explains the widespread epidemic and sporadic outbreaks. the high genetic variability is due to error of RNA-dependent RNA polymerase and also to recombination [4,5]. Great genetic diversity is favored also by co-circulation and co-infection [6]. Recombination plays a key role, since it can lead to forms capable of escaping the immune system, overcoming specific antibody selection in the infected host [7].

## 2. Epidemiology

EVs are among the most common viruses infecting humans and are responsible for an estimated 10 to 15 million cases of symptomatic infections every year in the US [8]. Data from the US show furthermore that children less than one year of age may account for about 40% of EVs infections in patients with known age [9]. Verboon-Maciolek et al. estimated an incidence of EVs infection in the neonatal period of 26 cases per 100,000 live births in the Netherlands [10]. 

Non-polio EVs show seasonal patterns of incidence. In temperate areas, EVs are characteristically found in the summer and early autumn, although outbreaks may continue into the winter; in tropical climates, circulation tends to be year-round or associated with the rainy season. In Europe, approximately 50–65% of infections affecting infants occur from April to October. There are frequent fluctuations in predominant EVs serotypes, with some serotypes showing periods of relative quiescence followed by extensive outbreaks. Variation by location is an important characteristic of EVs epidemiology [11,12,13,14,15,16]. Table 2 shows the extreme variability of predominant serotypes in neonates and infants according to different studies. 

EVs outbreaks may affect small groups or communities (such as schools or hospital wards) or may become widespread at a regional, national, or even international level [17]. Greater genotypic heterogeneity is observed in early infancy, possibly because the immune system is more mature in older children. Echovirus 11 was the most frequent genotype isolated from children under five years of age in northern Italy between 2010 and 2014; Echovirus 11 was associated with severe disease (acute hepatitis, meningitis, acute flaccid paralysis) in 75% of cases, particularly in neonates [18]. In recent years, authors reported that some European countries are experiencing a rise of Echovirus 30 infections, a serotype that usually causes central nervous system infections, particularly among neonates [19].

EVs spread from person to person by the fecal-oral and by the respiratory routes but indirect transmission may also occur via different routes, including contaminated water, food and fomites [20]. Neonatal EVs infections can be acquired antenatally (transplacentally or via an ascending route), intrapartum (usually with clinical presentation from two to seven days of life) and postnatally (usually by family members but sometimes from healthcare workers) [3,17]. Inadequate hand washing can contribute to the spread of EVs infections in the neonatal intensive care unit (NICU); however, vertically acquired infections are usually more severe than horizontally acquired [21]. A recent study on neonates and young infants in Japan by Izumita reported EVs positive stool samples in 91% of siblings and 42% of parents. Most of them (45% of siblings and 85% of parents) were asymptomatic [22]. 

EVs infections may be also transmitted via breast milk although we emphasize the protective role of breast milk against viral infections, particularly in the first year of life. In a prospective study 150 infants were followed during the first year of age; investigators found that EVs infections were less common among those who were exclusively breastfed for at least 2 weeks (0.38 vs 0.59 infections per child, *p* = 0.04) [23].

Classically reported risk factors for severe neonatal EVs infections include: preterm birth, maternal viral infections in the peripartum, onset of symptoms in the first week of life, and lack of specific antibodies [24,25,26,27]. A retrospective study was carried out in Australia between 2008 and 2012; among 109 cases of EVs meningoencephalitis (all age groups) the higher incidence was found in the pediatric age, particularly under three months of age (46 out of 109 cases, 42% of total cases); among these 46 infants, 26 (56%) were newborns (under one month of age) and 9 (20%) were preterm born [28].

## 3. Pathogenesis

Following oral or respiratory transmission, EVs reach the pharynx and the alimentary tract and subsequently spread to regional lymphoid tissues (tonsils, Peyer patches, and regional lymph nodes). The viremia occurs in a few days; EVs spread to the reticuloendothelial system, including liver, spleen, bone marrow and more distant lymph nodes. Host immune responses may limit the viral replication, preventing the spread of infection to other sites (subclinical infection). In contrast, the replication in the reticuloendothelial system may lead to a major, secondary viremia (after 3 to 7 days), which involves the CNS, heart and skin, and overlaps to the onset of symptoms. The viral load in secondary sites usually reduces after approximately seven days. Antibodies are the most important immune defense against EVs infections, and viremia recovers when type-specific neutralizing antibody levels increase [29].

Mutations in the structural region of the capsid, which have been observed in EVs, can alter the antigenicity of each strains and the consequent antibody response, increasing the ability of the virus to evade the immune system [7,30].

**Figure 1:**
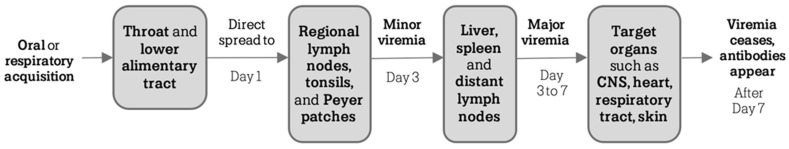
Pathogenetic stages of EVs infection [3].

Figure 1 details the stages of the pathogenetic process of EVs infection.

## 4. Clinical Findings

### 4.1. Abortion, Stillbirths and Intrauterine Infections

In a Swedish serological investigation, IgM antibodies against Coxsackievirus group B were found in 42% of pregnant women with abortion as compared with 18% of controls [31]. Several case reports describe isolation of EVs from amniotic fluid, often in association with maternal symptoms suggestive of chorioamnionitis and with fetal demise. In some cases, the viral agent has been identified in the myocardium, brain, liver and placenta of stillborn infants [3]. Coxsackievirus infections during pregnancy have been associated with congenital anomalies (such as urogenital anomalies, cardiovascular defects) in serological studies [32]. 

### 4.2. Clinical Manifestations in Neonates and Infants

The vast majority of EVs infections are asymptomatic and yet may serve as significant sources for spread of infection. The early epidemiological study by Jenista et al. carried out in neonates from birth to day 28 of life found an asymptomatic carriage in 59 (79%) of 75 EVs infected neonates [33]. Symptomatic infections are more common in young children. Clinical manifestations range from minor febrile illness to severe conditions, sometimes fatal. Infections are usually more severe in neonates and young infants, especially under 1 year of age. Neonatal symptoms may occur as early as Day 1 of life, and severe disease usually occurs within the first two weeks of life [34]. Most have a benign course and fever resolves in an average of 3 days while additional symptoms usually recover within one week [17,35]. History includes a maternal viral illness preceding or immediately following delivery in approximately 60% of cases, with fever, respiratory findings, and/or abdominal pain. These symptoms are also commonly found in other family members [36].

Risk factors and clinical features associated with severe disease include absence of neutralizing antibody to the infecting serotype, maternal illness prior to or at delivery, prematurity, onset of illness within the first few days of life, severe hepatitis, multi-organ failure. Severe disease is also associated with specific infecting EVs serotypes (such as Group B coxsackieviruses and echovirus 11) [26,27,37,38]. A Chinese systematic review, that includes 66 articles and 237 cases of severe neonatal EVs infections (from 2000 to 2020), reports different rates of hepatitis or coagulopathy (46%), myocarditis (37%), meningoencephalitis (11%), other complications (6%) such as hemophagocytic lymphohistiocytosis and pulmonary hemorrhage [39]. Table 3 reports the incidence of main clinical findings in EVs infected neonates and infants’ populations reported in recent literature. 

#### 4.2.1. Mild, Nonspecific Febrile Illness and Sepsis-like Illness

Nonspecific febrile illness is the most common finding in EVs infections. Fever occurs in most neonates (76 to 100%), sometimes with a biphasic pattern, and may last from 1 to 5 days (usually 3–4 days) [24]. A retrospective study conducted in Taiwan on a population of 146 young infants < 3 months of age with EVs infection reports non-specific febrile illness as the final diagnosis in approximately 30% of cases [38]. A prospective study carried out on a population of 353 infants < 90 days with sepsis-like symptoms identified EVs as the causative agent in 132 (37%) of cases [43]. Febrile illness may sometimes present with alarming symptoms, which must be differentiated from sepsis or bacterial meningitis. The disease usually begins abruptly with fever (ranging from 38° C to 39° C or higher), malaise, irritability, poor feeding, lethargy, diarrhea and/or vomiting. In addition, a rash, highly variable in nature, but usually macular or maculo-papular, is frequently present.

#### 4.2.2. Central Nervous System Involvement

Neonates show a higher risk of severe systemic illness, meningitis and meningoencephalitis. EVs meningitis is almost three-fold more frequent than bacterial meningitis and the estimated incidence in high-income countries is 12–19 cases per 100,000 population per year [44,45]. Non-polio EVs are the leading cause of viral meningitis, accounting for 85 to 95% of the cases in which an etiologic agent is identified [46,47,48]. Kadambari and colleagues found that EVs were responsible for the vast majority of meningoencephalitis reported in the UK and Ireland in infants aged less than 3 months with an estimated incidence of 79 cases/100,000 live births per year [16].

The term aseptic meningitis refers to a clinical syndrome of meningeal inflammation in which common bacterial agents cannot be identified in the cerebrospinal fluid (CSF). The initial clinical findings in neonatal meningitis are similar to those in sepsis-like illness. CSF often shows pleocytosis (frequently < 500 but occasionally higher than 1000 WBCs/μL); polymorphonuclear cells are often predominant in the first 48 h before becoming mostly mononuclear [49,50,51]. Cerebrospinal fluid can have normal parameters despite positive viral culture or PCR results, particularly in the first few months of life and early after illness onset. Wide variations in all parameters, however, are the rule, even during an epidemic involving a single serotype. Therefore, initial CSF parameters may not be helpful in ruling out bacterial infections of the central nervous system or confirming a viral etiology. 

EVs are responsible for 10–20% of encephalitis with an identified cause [52]. Encephalitis with parenchymal brain involvement is less frequent than meningitis. Encephalitis is suggested by extreme lethargy that may progress to altered levels of consciousness. Seizures may occur and a bulging fontanelle may sometimes be evident. Up to 40% of cases may present with focal findings (seizures, myoclonus and hemichorea), whereas MRI study confirms the white matter damage [39,53,54,55]. 

#### 4.2.3. Cardiac Involvement

Myocarditis is an inflammation of the myocardium associated with damage that is unrelated to an ischemic injury. EVs account for approximately 25–35% of cases of myocarditis and pericarditis with proven cause. Group B coxsackieviruses are the predominant causative agents of acute EVs myocarditis (specially CVB 1–5), although Coxsackie A and echoviruses also may be causative [56,57]. However, only a very small proportion of EVs infections result in overt cardiac involvement. 

EVs damage the heart primarily via direct lysis of infected myocytes, but immune-mediated mechanisms may also play a role. Neonatal EVs myocarditis may present abruptly with arrhythmias, congestive heart failure and cardiogenic shock and can lead to multi organ failure. Fever, malaise, and anorexia are frequently part of the initial clinical presentation. Furthermore, many patients show signs of respiratory distress and cyanosis. Cardiac findings normally include transitory systolic murmur and electrocardiographic changes (such as supraventricular tachycardia, ST segment depression, and T wave abnormalities). Echocardiography shows cardiomegaly, reduced contractility, low ejection fraction, and sometimes pericardial effusion. Myocardial enzyme serum concentration may be raised. Chest radiography often demonstrates cardiac enlargement [27,58]. 

Myocarditis is often accompanied by disseminated viral infection involving the central nervous system, liver, pancreas and adrenal glands. Mortality may be as high as 30–50% [39]. EVs myocarditis may lead to cardiovascular collapse refractory to conventional resuscitation measures. When cardiovascular failure is present, progression is usually rapid, often within two days from the onset of illness [59,60]. However, in approximately a third of patients, cardiovascular collapse may occur several days after the onset of EVs infection. In a few cases, neonates critically ill with EVs myocarditis have been supported with extracorporeal membrane oxygenation (ECMO) as a bridge to cardiopulmonary recovery [61].

#### 4.2.4. Gastrointestinal Manifestations and Acute Hepatitis

Gastrointestinal signs and symptoms are frequently present, but they are rarely isolated. Among 27 neonates with EVs infection, vomiting, abdominal distension and diarrhoea were reported in 33%, 70% and 81% of cases, respectively. Moreover, three of 27 had necrotizing enterocolitis [27]. 

Acute hepatitis and coagulopathy are serious manifestations of neonatal infections, often associated with Echovirus infections (types 5, 11, 6, 7, 9, 14, 17,19, 21) and Coxsackievirus B infections [62,63]. Findings that are commonly related to this condition include hepatomegaly, jaundice and laboratory abnormalities, such as coagulopathy, elevated transaminases, and hyperbilirubinemia. Coagulopathy is characterized by thrombocytopenia, clotting time prolongation, fibrin split products and bleeding diathesis. Acute hepatic necrosis and liver failure may complicate the course [64]. Among 42 cases of EVs hepatitis and coagulopathy, a fatal outcome was reported in 10 (24%) cases. Most significant factors associated with mortality were higher total bilirubin levels and a concomitant myocarditis [38].

#### 4.2.5. Respiratory Illnesses

Respiratory manifestations are usually overshadowed by other clinical findings of neonatal EVs infections. Morens and colleagues report only 7% of 338 EVs infections in infants less than two months of age classified as respiratory illnesses [24]. Respiratory symptoms that may be observed in neonatal EVs infections are cough, wheezing, rhinorrhea, tachypnea [17]. Clusters of severe respiratory disease have been observed with multiple lineages of enterovirus D68 [65,66]. Rare respiratory complications of neonatal EVs infections are pulmonary hemorrhage, persistent pulmonary hypertension; is also described a case of pulmonary hypoplasia in a 38 weeks gestational age newborn with a vertical infection by Echovirus 11 [39]. Life-threatening pulmonary edema, hemorrhage, and interstitial pneumonitis may complicate encephalitis caused by EV-A71 [67,68,69].

#### 4.2.6. Exanthems and Hand-Foot-Mouth Disease

Infants with EVs infections can develop skin lesions which are usually macular or maculopapular eruptions, but petechial lesions occasionally occur. Rashes have occurred with many serotypes, such as coxsackieviruses B1, B3 and B5, and echoviruses 4, 5, 6, 9, 11, 16, 17, 18, and 21 [35]. Of 27 infants studied by Lake, 41% had rash [34]. Abzug and colleagues reported 38% of 29 neonates aged 0 to 14 days with EV infection had rashes [36].

Hand-foot-and-mouth disease (HFMD) is a common rash syndrome, caused by human enterovirus species A (HEV-A). The most important serotypes are coxsackievirus A16 and EV-A71 [70,71]. Scattered vesicular lesions occur in the posterior pharynx, the buccal mucosa, tongue, palate, gingiva and lips; these vesicles ulcerate readily, leaving shallow lesions with red areolae. Frequently also appear sparse grayish maculopapular or pustular vesicles (3 to 5 mm in diameter, surrounded by erythematous areolae) on the dorsum of the fingers, particularly in periungual areas, and on the margins of heels. Lesions on the palms, soles of the feet, buttocks, groin, and sometimes knees and elbows may appear occasionally [72]. Xu reported 16 cases of neonatal HFMD with coxsackievirus A6 infection between 2016 and 2017 in Shanghai, China [73].

## 5. Diagnosis

A specific diagnosis of EVs infections implicates detection of the virus in biological samples. Confirmation of the diagnosis can be important in reducing hospitalization, inappropriate antibiotic use, and additional diagnostic testing often performed to rule out other conditions [74,75,76,77]. Because speed, specific and sensitive, amplification of viral RNA through reverse transcription polymerase chain reaction (RT-PCR) has already replaced viral isolation in cell culture. RT-PCR for primary diagnosis targets the 5′ untranslated region (5′UTR) [78]. 

RT-PCR is proven to be useful in the identification of EVs in CSF, plasma, throat, tracheal, rectal, stool, and urine specimens. By using blood and CSF RT-PCR, more than 25% of infants admitted to the hospital for suspected sepsis over a year period tested EVs positive [79]. Therefore, the workup for febrile neonates admitted to hospital for suspected sepsis or meningitis should include EVs RT-PCR testing in blood and CSF samples. A recent prospective study was conducted in newborn, infants and children to assess the sensitivity of blood EVs RT-PCR test in the workup of fever without source, sepsis-like illness and suspected meningitis. Among 71 EVs infected neonates, RT-PCR confirmed EVs more commonly in plasma (99% of cases) with respect to CSF samples (87% of cases, *p* = 0.01) [40]. Among 128 infants aged 0 to 90 days who underwent sepsis evaluation, the duration of empiric antibiotic treatment was significantly reduced by the use of EVs RT-PCR. EVs positive infants showed a shorter duration of empiric antibiotic therapy compared to EVs negative patients (median duration 18 h vs 48 h, *p* < 0.0001) and EVs RT-PCR untested patients (median of 18 h vs 48 h, *p* < 0.001) [80]. Moreover, investigators from the European Non-Polio Enterovirus Network recommend collecting respiratory and stool samples in suspected CNS EVs infection [81].

RT-PCR represents a valuable diagnostic tool but requires expensive instrumentation not available in all facilities. Recently, Daskou and colleagues developed a colorimetric loop-mediated isothermal amplification (LAMP) assay, which demonstrated high sensitivity and specificity, with the advantage of faster time and lower costs in comparison to PCR [82].

The gold standard for EVs typing is represented by sequencing of the VP1 capsid protein gene. Sequencing multiple parts of the full-length genome is used to identify the emergence of recombinant EVs variants [81]. From a future perspective, a promising technique is represented by metagenomic next-generation sequencing, which offers multiple advantages including the identification of virulence determinants and the prediction of resistance to antivirals [83].

Serologic methods implicate the detection of a rise in antibody titers after infection. 

Nevertheless, the need of collecting both an acute and a convalescent serum specimen makes serologic diagnosis impractical and time consuming in the clinical setting [3].

Further potentially severe viral or bacterial infections should be ruled out. When plasma or CSF test EVs RT-PCR positive a concomitant bacterial infection is unlikely. In a recent prospective multicentre study only 6% (22 of 360) of neonates and children with EVs meningitis or sepsis were found to have concomitant bacterial infection. Most of them had a urinary tract infection and none had a bacterial sepsis or meningitis [40]. This findings was consistent with previous studies [79,84,85]. Therefore, empiric antibiotic treatment may be discontinued in most cases when EVs is confirmed in samples [86]. 

Human parechoviruses must be considered in the differential diagnosis of EVs infection because they are recognized as an important cause of disease in neonates and infants with a similar clinical spectrum. Since they represent a distinct genus, a specific PCR assay must be performed to identify parechovirus infections [87,88]. 

## 6. Complications and Outcome

The outcome of EVs infections is usually excellent, although in early studies case fatalities reached up to 80% [26]. In the previously mentioned systematic review by Zhang concerning life-threatening infection in neonates, investigators reported a case-fatality rate of approximately 30%. Morbidity and mortality were often associated with myocarditis and liver involvement in neonates or immunocompromised children [39]. 

Hepatic, cardiac or neurologic sequelae may occur after EVs infections. Hepatic injury may last up to infancy, although improvement usually occurs in surviving infants. Severe hepatitis can lead to cirrhosis and chronic hepatic failure. 

The overall acute mortality after EVs myocarditis may reach 4%. In most cases, a full recovery usually occurs after months. Nevertheless, heart sequelae (chronic dilated cardiomyopathy, calcific myocarditis, ventricular micro-aneurysms, chronic heart failure, dysrhythmias, or constrictive pericarditis) may sometimes occur [58,61]. 

Some studies on the outcome of central nervous system infection have reported long-term morbidity, including spasticity, hypotonicity, motor weakness, intellectual deficits, seizure disorders, hydrocephaly, microcephaly, ocular abnormalities and delayed speech and language development [52,89]. Nevertheless, there are a lack of large studies detailing the neurodevelopmental outcome of EVs infections.

## 7. Treatment

Potential antiviral drugs that target various stages of the viral replication cycle are currently being evaluated in-vitro and in animal models. In some cases, Phase I and II in humans have been carried out. 

At present, the most advanced antiviral treatment relies on two antiviral agents (*Pleconaril* and *Pocapavir*). Both integrate into the capsid and inhibit viral attachment and uncoating. A recent systematic review evaluated 66 studies concerning neonatal severe EVs infections. *Pleconaril* and *Pocapavir* were administered in 5.9% and 1.3% of 237 severe infections, respectively [39]. 

*Pleconaril* is at present one of the most studied antiviral treatment for severe EVs infections in neonates. A recent double-blind placebo-controlled study enrolled 61 neonates with suspected or proven EVs infection aged less than 2 weeks, of which 43 received *Pleconaril* and 18 were given placebo. However, the study failed to demonstrate a faster viral clearance after treatment as compared to untreated neonates and no increase of survival was demonstrated, although adverse events were rare [90].

In-vitro tests show that *Pocapavir* (V-073) has antiviral activity against some clinical isolates of non-polio EVs. However, *Pocapavir* has been administered in only four neonates. The first report concerns a neonate with coagulopathy, cardiopulmonary failure and coxsackievirus B3 infection. The newborn improved after treatment without any apparent adverse effects [91]. The second study reports the use of *Pocapavir* and intravenous immunoglobulins (IVIG) in a late preterm female presenting at 14 days of life with EVs myocarditis and hepatitis. The neonate had improved liver function after antiviral treatment, despite persistence of a dilated cardiomyopathy [92]. Finally, *Pocapavir* use was reported in two monochorionic diamniotic twins who both presented with EVs myocarditis and cardiogenic shock on Day 12 of life. The patients in this report gradually improved and showed no adverse effect related to *Pocapavir* administration. Nevertheless, the contribution of each intervention is difficult to assess since they both required ECMO and received IVIG prior to *Pocapavir* treatment [93].

Additional treatments have been tested in animal models and in-vitro [94]. Alpha interferon, lactoferrin and ribavirin have shown antiviral activity against EV-A71, although there are no studies on neonates. Among them, ribavirin also plays an important role in studies concerning the evolution of EVs under strong mutagenic pressure, with potential implications for the development of new vaccines. In fact, restricting the quasispecies diversity of a viral population provides a new approach to engineering live attenuated vaccines for RNA viruses and ribavirin represents a tool to isolate high-fidelity variants of EVs [95,96].

Antibodies are a key host defense against EVs infections and IVIG contain variable amounts of neutralizing antibodies to EVs. Therefore, IVIG are sometimes administered for treating severe EVs infections, although evidence of their effectiveness is poor. Abzug and colleagues carried out a randomized clinical trial and IVIG were administered to 9 of 16 neonates. Treated neonates had a faster clearance of viremia and viruria, but the study was too small to demonstrate any significant effect on clinical outcomes [97]. A retrospective study investigated 41 neonates with severe EVs hepatitis and coagulopathy treated with IVIG. The rate of survival was high (93%) when IVIG were given within 3 days from the onset of symptoms. However, neonates were not randomized making it difficult to compare early and late treatments [98]. 

Overall, at present there is a lack of strong evidence to support antiviral treatments or IVIG in neonates. Most studies enroll very few neonates or are single case reports. Therefore, in a clinical setting, the only treatment is mainly supportive. Careful clinical surveillance is important since neonates may worsen after 2 or 3 days from the onset of symptoms.

### Prevention Strategies

Standard hygiene measures, particularly hand washing, play an important role in preventing nosocomial transmission of EVs infections. Cohorting is effective in controlling virus spread when outbreaks occur within the nursery or the NICU [99].

Enterovirus A71 is the main causative agents of HFMD and large outbreaks caused by EV-A71, especially in the Asia–Pacific region, may sometimes lead to severe acute neurologic disease (such as encephalitis, acute flaccid paralysis and polio-like syndrome) and life-threatening cardiopulmonary failures [71,100,101]. Due to the socioeconomic impact of EV-A71 infection, great interest has been directed towards the development of an effective vaccine. Five formalin-inactivated EV-A71 vaccines, evaluated in human clinical trials in China, Taiwan, and Singapore, were found to be safe and to stimulate strong neutralizing antibody responses against EV-A71 [102,103,104]. A Phase IV study was carried out in over 150,000 children. The incidence of EV-A71 infection decreased after vaccine administration. Nearly 90% of hand foot and mouth diseases and infection-associated hospital admissions were prevented, especially among children aged 6 to 35 months [105].

## 8. Conclusions

EVs infections are common in children. Given the extreme variability of predominant serotypes in different geographical areas and in different years, a close molecular epidemiological surveillance would be desirable, in order to control the spread of new outbreaks through appropriate preventive measures. Infections can be severe, especially in the first days of life, and may lead to death, long-term sequelae, and neurodevelopmental delay. Currently, the treatment of severe infections is mainly supportive, given the lack of strong evidence on the use of antivirals or IVIG. In milder cases, the impact on neurodevelopmental outcome is less clear, due to the lack of prospective studies in large populations.

## Figures and Tables

**Table 1:** Classification of Enteroviruses.

Species	Serotypes
Enterovirus A (HEV-A)	Coxsackievirus A2–8, A10, A12, A14, A16 Enterovirus A71, A76, A89–91, A114, A119–121
Enterovirus B (HEV-B)	Coxsackievirus A9 Coxsackievirus B1–6 Echovirus 1–9, 11–21, 24–27, 29–33 Enterovirus B69, B73–75, B77–88, B93, B97, B98, B100, B101, B106, B107
Enterovirus C (HEV-C)	Poliovirus 1-3 Coxsackievirus A1, A11, A13, A17, A19–22, A24 Enterovirus C95, C96, C99, C102, C104, C105, C109, C113, C116–118
Enterovirus D (HEV-D)	Enterovirus D68, D70, D94, D111

Adapted from reference [1,2].

**Table 2:** Epidemiology of EVs infection in newborn and infants.

Author-Year	Verboon-Maciolek-2008	Rodà-2015	Cabrerizo-2015	Lv-2016	Cabrerizo-2017	Kadambari-2019
Reference	[11]	[12]	[13]	[14]	[15]	[16]
Country	Netherlands	Spain	Spain	China	Spain	UK-Ireland
Single center/multicenter	Single centre	Single centre	Multicentre	Single centre	Multicentre	Multicentre
Period in study	Jan 1994–Dec 2016	Mar 2010–Dec 2010	Jan 2013–Dec 2013	Mar 2011–Sep 2012	Jan 2010–Dec 2013	Jul 2014–Jul 2015
Age at diagnosis (d)	0–30	0–90	0–30	0–30	0–30	0–90
Inclusion criteria	EVs confirmed cases	Fever > 38 °C	Fever > 38 °C, ME, CS	Fever > 38 °C	Fever > 38 °C, ME CS, HFMD, AE	ME
positive/tested (%)	21/21 (100)	195/699 (28)	32/84 (38)	131/334 (39)	249/1430 (17)	668/668 (100)
M/F	14/7	114/81	17/15	85/46		
Predominant serotypes (%)	CV-B5 (33)	E-5 (21.1)	E-18 (24)	CV-B1 (47)	E-5	E-9 (20)
	E-11 (9.5)	E-11 (11.8)	E-3 (14)	E-30 (35)	E-11	E-18 (12)
	E-6 (9.5)	E-21 (7.2)	E-5 (14)	CV-B3 (10)	CV-B4	CV-B5 (8)
	CV-A9 (9.5)	E-25 (7.2)	CV-B3 (10)	E-16 (4)		CV-B4 (7)
	Other (28.6)	CV-B4 (6.4)	Other (38)			E-25 (6)
Seasonal Period (%)	Jun–Oct (62)	Mar–Aug (73)	Apr–Jul (66)	Mar–Aug (100)	Apr–Jun (50)	Summer (ns)

ME, meningoencephalitis; CS, clinical sepsis; HFMD, hand foot and mouth disease; AE, atypical exanthem; CV, coxsackievirus; E, Echovirus; ns, not specified; M/F, male/female.

**Table 3:** Most frequent clinical findings of EVs infection in newborn and infants.

Author (Year)	Verboon- Maciolek (2008)	March (2014)	Rodà (2015)	Lv (2016)	Lafolie (2018)	Berardi (2019)	Kadambari (2019)	Chen (2021)
Reference	[11]	[28]	[12]	[14]	[40]	[41]	[16]	[42]
Age at diagnosis (d)	0–30	0–30	0–90	0–30	0–30	0–90	0–90	0–30
Inclusion criteria	EVs confirmed cases	Positive CSF PCR	Fever > 38 °C	Fever > 38 °C	Fever > 38 °C, CS, suspected ME	Positive plasma or CSF PCR	ME	Positive throat swab or CSF PCR
Symptoms								
Fever (%)	76	83	100	100	100	81.8	85	100
Poor feeding (%)	ns	40	ns	16.8	28	31.8	54	ns
GI symptoms (%)	52	ns	37.5	52.6	10	29.5	ns	0
Respiratory symptoms (%)	52	ns	21.1	36.6	14	ns	12	0
Irritability (%)	38	40	26.3	13.7	62	59.1	66	26
Lethargy (%)	ns	ns	ns	2.3	ns	ns	36	0
Rash (%)	23	17	13.2	26	6	2.3	24	39.1
Seizure (%)	42	2.2	ns	1.5	0	6.8	ns	0

ME, meningoencephalitis; CS, clinical sepsis; ns, not specified; GI, gastrointestinal; CSF, cerebrospinal fluid; PCR, polymerase chain reaction.
